# Clinical features and major bleeding predictors for 161 fatal cases of COVID-19: A retrospective observational study

**DOI:** 10.17305/bjbms.2021.6577

**Published:** 2021-11-22

**Authors:** Gokhan Alici, Hasan Ali Barman, Ramazan Asoglu, Adem Atici, Atike Nazli Akciger, Omer Sit, Omer Dogan, Yucel Yavuz, Songul Borahan, Omer Genc, Baris Gungor

**Affiliations:** 1 Department of Cardiology, University of Health Sciences, Okmeydani Training and Research Hospital, Istanbul, Turkey,; 2 Department of Cardiology, Istanbul University – Cerrahpasa, Institute of Cardiology, Istanbul, Turkey,; 3 Department of Cardiology, Adiyaman Training and Research Hospital, Adiyaman, Turkey,; 4 Department of Cardiology, Istanbul Medeniyet University, Faculty of Medicine, Goztepe Training and Research Hospital, Istanbul, Turkey,; 5 Department of Anesthesiology and Intensive Care, University of Health Sciences, Okmeydani Training and Research Hospital, Istanbul, Turkey,; 6 Department of Cardiology, Agri Training and Research Hospital, Agri, Turkey,; 7 Department of Cardiology, University of Health Sciences, Dr. Siyami Ersek Training and Research Hospital, Istanbul, Turkey

**Keywords:** Acute respiratory distress syndrome, cardiac injury, COVID-19, major bleeding, mortality

## Abstract

The aim of this study was to investigate the patient characteristics and laboratory parameters for coronavirus disease-2019 (COVID-19) non-survivors as well as to find risk factors for major bleeding complications. For this retrospective study, the data of patients who died of COVID-19 in our intensive care unit were collected in the period of March 20, 2020-April 30, 2020. D-dimer, platelet count, C-reactive protein (CRP), high-sensitivity Troponin I (hs-TnI), and international normalized ratio (INR) levels were recorded on the 1^st^, 5^th^, and 10^th^ days of hospitalization to investigate the possible correlation of laboratory parameter changes with in-hospital events. A total of 161 non-survivors patients with COVID-19 were included in the study. The median age was 69.8 ± 10.9 years, and 95 (59%) of the population were male. Lung-related complications were the most common in-hospital complications. Patients with COVID-19 had in-hospital complications such as major bleeding (39%), hemoptysis (14%), disseminated intravascular coagulation (DIC) (13%), liver failure (21%), acute respiratory distress syndrome (ARDS) (85%), acute kidney injury (AKI) (40%), and myocardial injury (70%). A multiple logistics regression analysis determined that age, hypertension (HT), diabetes mellitus (DM), use of acetylsalicylic acid or low-molecular-weight heparin (LMWH), hemoglobin, D-dimer, INR, and AKI were independent predictors of major bleeding. Our results showed that a high proportion of COVID-19 non-survivors suffered from major bleeding complications.

## INTRODUCTION

The outbreak of severe acute respiratory syndrome coronavirus-2 (SARS-CoV-2) infection has been named as coronavirus disease 2019 (COVID-19) [1]. The SARS-CoV-2 infection causes pulmonary and/or systemic inflammation and leads to multiple organ failure, especially in high-risk patients [2]. SARS-CoV-2 binds to the angiotensin-converting enzyme-2 (ACE-2) receptor, which is mainly expressed in lung tissue and infects multiple cells and tissues in the human body [3,4]. SARS-CoV-2 infection may cause acute respiratory distress syndrome (ARDS), myocardial damage, and heart failure [5,6]. COVID-19 patients usually present with mild symptoms (no pneumonia or mild pneumonia) (81%). On the contrary, few COVID-19 patients (about 14%) might have severe symptoms, respiratory failure, septic shock, and/or multiple organ failure [7]. Major risk factors for COVID-19-related mortality were advanced age, male gender, hypertension (HT), diabetes mellitus (DM), cardiovascular disease (CVD), and cerebrovascular diseases [8]. COVID-19 patients might have a myocardial injury, myocarditis, arrhythmias, and thromboembolic events without having risk factors [9,10].

Severe COVID-19 infection is associated with disruption of normal hemostasis, elevated D-dimer levels, and a systemic procoagulant course leading to thromboembolism [11]. Patients in critical condition due to COVID-19 are at high risk for hypercoagulation and anticoagulation therapy is recommended for these patients [12]. COVID-19 patients are predisposed to life-threatening major bleeding due to systemic anticoagulation [13-15]. Regardless of the pharmacological agent used, the risk of major bleeding in the critical group of COVID-19 patients receiving thromboprophylaxis is estimated to be 5.6% [16]. The assessment of clinical features and bleeding predictors of non-survivors COVID-19 patients may yield additional data for risk stratification and guidance of treatment.

We aimed to investigate the patient characteristics and laboratory parameters of COVID-19 non-survivors and find risk factors for major bleeding complications.

## MATERIALS AND METHODS

### Study population

The study included the patients who were non-survivors due to COVID-19 in our clinic between March 20, 2020, and April 30, 2020. All patients were diagnosed with COVID-19 using real-time reverse transcription-polymerase chain reaction (rRT-PCR). Patients whose diagnosis was not confirmed by RT-PCR, those who deceased before hospital admission, and patients aged under 18 years were excluded from the study. Demographic characteristics including age, gender, smoking status, and history of hyperlipidemia, HT, and DM, and in-hospital events were obtained from medical records in a retrospective manner. Laboratory parameters including urea, creatinine, sodium, potassium, glucose, high-sensitivity Troponin I (hs-TnI), D-dimer, creatine kinase-MB, hemoglobin, white blood cell (WBC), procalcitonin, and C-reactive protein (CRP) were measured from the blood samples obtained on admission and follow-up. In addition, D-dimer, platelet count, CRP, troponin, and international normalized ratio (INR) levels were recorded on the 1^st^, 5^th^, and 10^th^ days of hospitalization to investigate the possible correlation of laboratory parameter changes with in-hospital events.

The presence of SARS-CoV-2 RNA was detected by rRT-PCR in the Ministry of Health Public Health Microbiology Reference Laboratory after obtaining oropharyngeal and nasal specimens using the same swab and placing the swab on the same transport medium. Routine confirmation of the diagnosis of COVID-19 was performed based on the detection of unique sequences of virus RNA by nucleic acid amplification tests such as rRT-PCR with confirmation by nucleic acid sequencing when necessary. The guidelines for COVID-19, which was prepared by the Ministry of Health, were implemented and medications were used in patients. Low-molecular-weight heparin (LMWH) was used for thromboprophylaxis in patients without active bleeding. ASA was continued in patients who had previously used it for various reasons such as coronary artery disease (CAD) or peripheral arterial disease.

### Definitions

Myocardial injury was defined as a troponin value exceeding the upper reference limit (URL, 99%) according to the myocardial infarction guideline updated in 2018 [17]. Acute kidney injury (AKI) was defined based on the kidney disease: Improving Global Outcomes definition [18]. Patients with a history of percutaneous coronary intervention or coronary artery bypass surgery were diagnosed as CAD. The severe group was defined as respiratory rate ≥30/minutes, and/or severe respiratory distress (dyspnea, use of extra respiratory muscles), and/or oxygen saturation in room air <90% (in the patient receiving oxygen PaO_2_/FiO_2_≤300), and/or, presence of any critical complications (shock, multiorgan dysfunction requiring admission to the intensive care unit (ICU), and/or any type of respiratory failure requiring mechanical respiratory support) [19]. ARDS is defined as new or increasing worsening in clinical condition, presence of pleural effusion, atelectasis, or bilateral infiltrates not defined as nodules, heart failure, or respiratory failure not explained by the volume load on the lung, hypoxemia as defined by a PaO_2_/FiO_2_ ratio ≤200 mmHg [20]. Major bleeding was defined as a fall in hemoglobin level of at least 2 g/dL (1.24 mmol/L) or requiring transfusion of at least two units of whole blood or red cells during hospitalization [21]. ISTH scoring system was used for the diagnosis of disseminated intravascular coagulation (DIC) [22]. Coagulopathy management in patients with COVID-19 has been conducted according to the national interim guideline on COVID-19, which is updated periodically [23].

### Ethical statement

This observational, retrospective study was conducted with the approval of the Okmeydani Training and Research Hospital Research Ethics Committee (no. 234/date: 16.06.2020) and was carried out by the principles of the Declaration of Helsinki.

### Statistical analysis

Kolmogorov–Smirnov test was used for the normal distribution analysis of the variables. Continuous variables were expressed as mean ± standard deviation and non-normally distributed variables were presented as median with interquartile range only due to the presence of abnormal distribution for all. Categorical variables were expressed as numbers (n) and percentages (%). Comparisons of continuous variables between groups were made using the t-test as a parametric test and the Mann–Whitney U-test as a non-parametric test. Chi-square test was used for the evaluation of categorical data. A two-tailed *p* < 0.05 was considered significant throughout the study. Univariate logistic regression analysis was used to determine significant independent clinical predictors of major bleeding. All variables with *p* < 0.05 in univariate analysis were included in a logistic regression model for multivariate analysis to determine independent predictors of major bleeding. The Kaplan–Meier method was used for survival analysis. A two-tailed *p<0.05* was considered statistically significant. Statistical evaluation was applied through the SPSS statistical program (version 25.0).

## RESULTS

Table 1 presents the demographic and clinical characteristics of 161 patients who were non-survivors due to COVID-19. The mean age was 71 (62-78) years and the female patients were older than male patients (*p* = 0.04). Following admission, 71% of the patients were hospitalized in the ICU. The mean hospitalization period was 10 days and no significant difference was found between male and female patients. The most common symptoms were fever and cough. However, no statistical difference was observed between the groups in terms of symptoms and findings. More than 50% of patients had at least one chronic disease, among which HT was the most common (60%), followed by DM (34%), CAD (33%), hyperlipidemia (21%), chronic obstructive pulmonary disease (COPD) (18%), chronic kidney disease (15%), atrial fibrillation (AF) (9%), and malignancies (8%). No significant difference was found between male and female patients with regard to the presence of chronic diseases. Female patients had more angiotensin II receptor blocker therapy compared to male patients (29% vs. 16%; *p*=0.04), while there was no significant difference between the genders with regard to other medications (*p* > 0.05). Lung-related complications were the most common in-hospital complications. Patients had in-hospital complications such as major bleeding (39%), hemoptysis (14%), DIC (13%), liver failure (21%), ARDS (85%), AKI (40%), and myocardial injury (70%). The prevalence of these complications was similar in both genders.

**TABLE 1:**
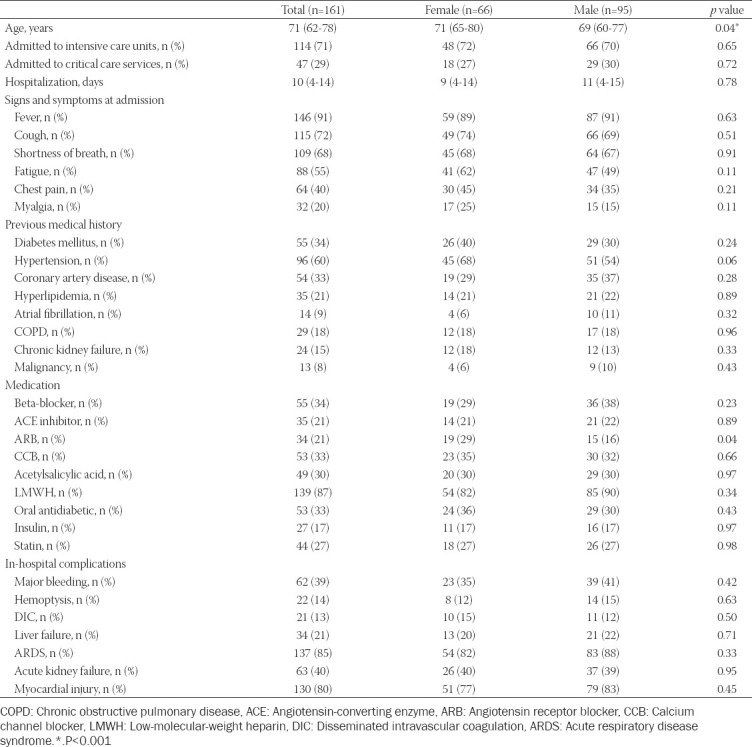
The demographic and clinical data of the study population

**FIGURE 1:**
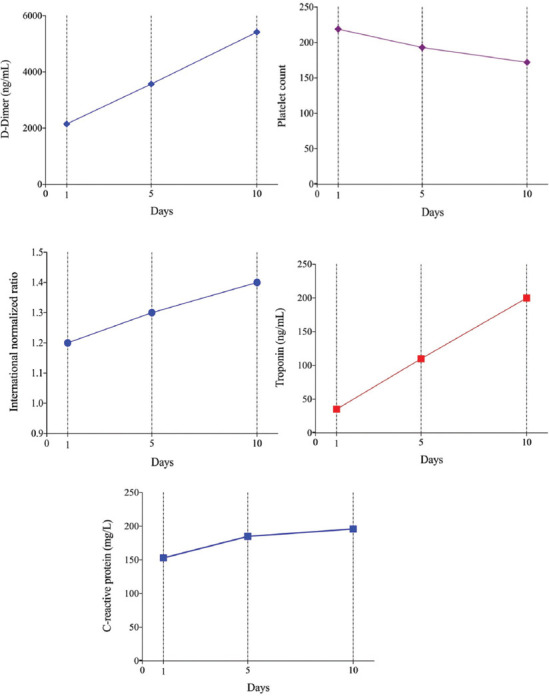
The platelet, D-dimer, international normalized ratio, C-reactive protein, and high-sensitivity troponin I levels of patients measured on the 1^st^, 5^th^, and 10^th^ days of hospitalization.

Table 2 presents the laboratory parameters measured at the time of admission. The glucose, urea, and creatinine levels were similar in both genders, whereas the sodium and potassium levels were significantly higher in women than in men. Lymphocyte levels were significantly lower in men compared to women. There was no significant difference between genders in terms of other blood parameters. Although the coagulation parameters of all patients were higher than the normal reference values, no significant difference was found between the genders with regard to prothrombin time (PT), activated partial PT, INR, and D-dimer levels (*p* > 0.05). Figure 1 presents the platelet, D-dimer, INR, CRP, and hs-TnI levels of patients measured on the 1^st^, 5^th^, and 10^th^ days of hospitalization. On the 5^th^ and 10^th^ days, the platelet levels decreased significantly while the D-dimer, INR, CRP, and hs-TnI levels increased significantly, compared to the 1^st^ day (Table 2 and Figure 1).

**TABLE 2:**
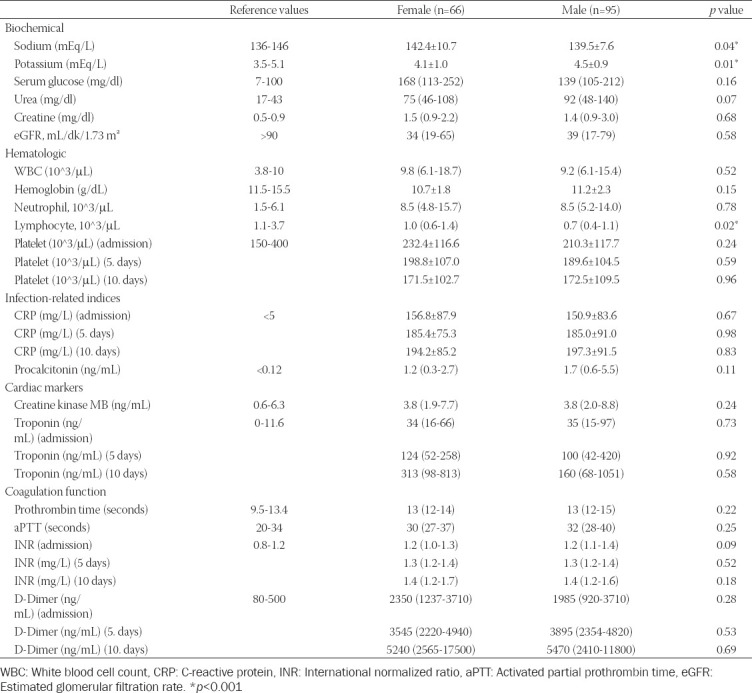
Laboratory parameters of study population

Major bleeding was observed in 62 (39%) of the patients. Patients with major bleeding (n = 62) and non-bleeding (n = 99) were compared in Table 3. The mean age of the non-bleeding group was older than the bleeding group (*p* = 0.03). The hospitalization time of the non-bleeding group was longer than the bleeding group (*p* < 0.001). There was no significant difference between the groups in terms of symptoms, signs, and the presence of chronic diseases. The rate of receiving LMWH therapy was higher in bleeding patients than in non-bleeding patients (100% vs. 78%; *p* < 0.001), whereas there was no significant difference between bleeding and non-bleeding patients with regard to other medications (*p* > 0.05). In the bleeding group compared to the non-bleeding group, hemoptysis (24% vs. 7%; *p* = 0.005), DIC (34% vs. 0%; *p* < 0.001), liver failure (39% vs. 10%; *p* < 0.001), ARDS (93% vs. 80%; *p* = 0.015), and acute kidney failure (48% vs. 33%; *p* < 0.001) were observed to be higher (Table 3).

**TABLE 3:**
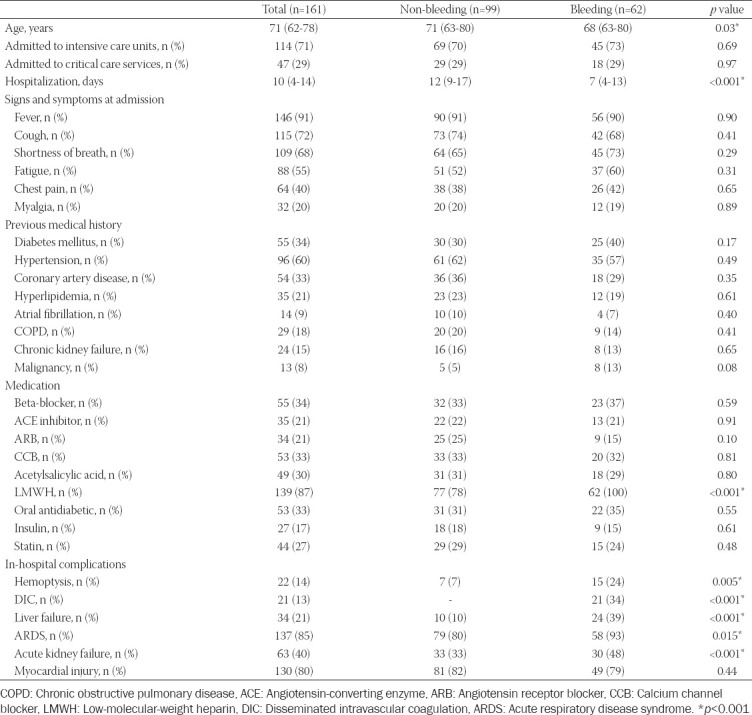
The demographic and clinical data of the study population with and without bleeding

Hematological and biochemical parameters were compared in both groups, serum potassium level (*p* = 0.01), WBC (*p* = 0.01), hemoglobin (*p* < 0.01), neutrophil (*p* = 0.02), platelet (5 days) (*p* = 0.02), and platelet (10 days) (*p* = 0.007) levels were higher in the non-bleeding group; troponin (admission) (*p* = 0.03), troponin (5 days) (*p* = 0.01), troponin (10 days) (*p* = 0.02), and INR (*p* = 0.007) levels observed high in the bleeding group (Table 4).

**TABLE 4:**
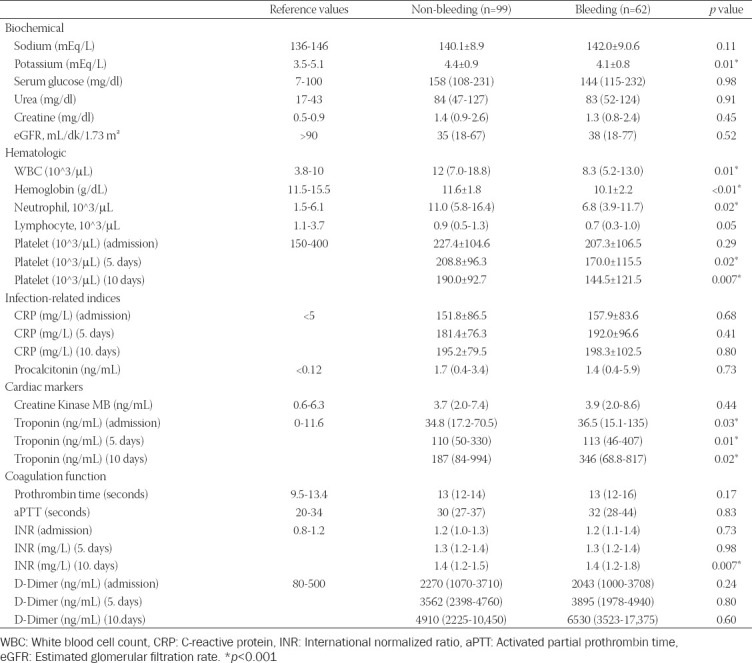
Laboratory parameters of study population with and without bleeding

**FIGURE 2:**
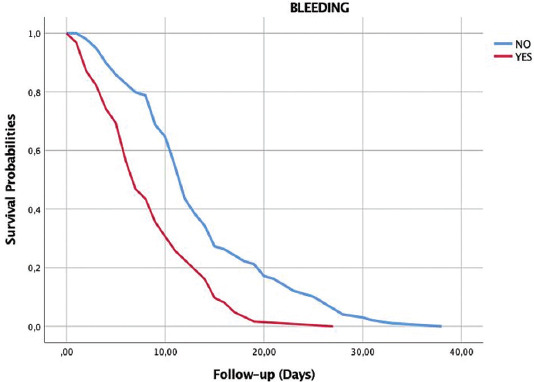
Survival analysis using the Kaplan–Meier method showed patients with and without bleeding.

Survival analysis was performed using the Kaplan–Meier method for patients with and without bleeding (Figure 2). Survival analysis showed that COVID-19 patients had a significantly increased risk of death in the weeks following ICU admission. Besides, as shown in the Kaplan–Meier survival analysis, the majority of deaths in our sample occurred within 20 days of admission (log-rank *p* < 0.001).

A multiple logistics regression analysis determined that age, HT, DM, use of acetylsalicylic acid (ASA) or LMWH, D-dimer, INR, and AKI were independent predictors of major bleeding (age, odds ratio [OR]: 1.052, *p* = 0.004; HT, OR: 3.271, *p* = 0.006; DM, OR: 1.672, *p* = 0.012; using of ASA, OR: 6.131, *p* < 0.001; using of LMWH, OR: 4.264, *p* < 0,001; D-dimer, OR: 1.002, *p* = 0.045; INR, OR: 1.999, *p* = 0.008; and AKI, OR: 1.792, *p* = 0.019) (Table 5).

**TABLE 5:**
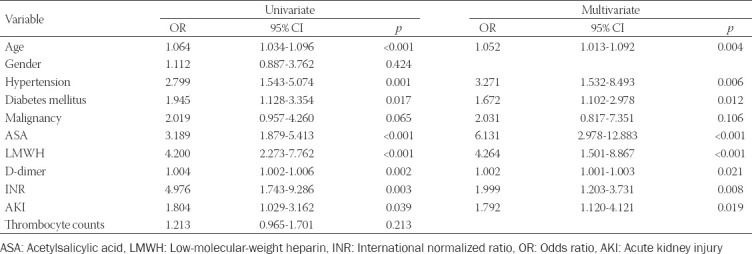
Univariate and multivariate logistics regression analysis on the risk factors associated with major bleeding in patients with non-survivor due to COVID-19

## DISCUSSION

The present study evaluated the clinical, laboratory features, and bleeding predictors of 161 non-survivors COVID-19 patients. The main findings of our study were as follows: (i) The most common presenting symptom was fever, lung-related complications were the most common complaints, (ii) the platelet levels decreased while the D-dimer, INR, and CRP levels increased during the follow-up period, and (iii) age, HT, DM, using of ASA or LMWH, hemoglobin, D-dimer, INR, and AKI were independently associated with major bleeding.

COVID-19 is characterized by hyperinflammation, cytokine storm, and elevated cardiac biomarkers. COVID-19 patients can present to the emergency service with a wide spectrum of symptoms ranging from asymptomatic or minimally symptomatic disease to pneumonia and severe ARDS [6,7,24]. The most common symptoms include fever (44-98%), fatigue, muscle pain, sore throat, dry cough (46-82%), shortness of breath (20-64%), and less commonly nausea, vomiting, and diarrhea (10%) [6]. Similarly, in our study, fever was the most common presenting symptom. Of the patients diagnosed with COVID-19, 6% of them exhibit a critical clinical picture characterized by ARDS, sepsis/septic shock, and/or multiple organ failure. These patients are hospitalized in ICU to prevent fatal complications. In patients requiring invasive mechanical ventilation, the mortality risk is remarkably high and the time from the onset of symptoms to death is 14 days [25]. The most common cause of death is respiratory failure (53%), followed by circulatory failure due to myocardial damage (7%), and respiratory and circulatory failure (33%) [26]. In our study, the mean hospitalization period was 10 days and lung-related complications were the most frequent complications.

Wu and McGoogan reported that patients with concomitant diseases had a higher risk of developing COVID-19 and also had higher mortality rates compared to the general population (10.5% in patients with CVD, 7.3% in diabetics, 6.3% in patients with COPD, 6% in patients with HT, and 5.6% in cancer patients) [8]. In our study, HT was the most common comorbidity (60%), followed by DM (34%), CAD (33%), hyperlipidemia (21%), COPD (18%), CKF (15%), AF (9%), and malignancies (8%).

It is shown that severe COVID-19 patients had an abnormality in coagulation parameters. In a retrospective cohort study in Turkey, we reported that a high D-dimer level (>1.41 g/L) was associated with in-hospital mortality (adjusted OR = 1.790, 95% CI: 1.078-2.972, *p* = 0.024) [27]. Another study observed that the patients who died had higher D-dimer levels and 71.4% of them were in the DIC clinic. In the same study, autopsy data suggested that arterial and microvascular thrombi may occur not only in the lungs but also in many other organs such as the heart and kidneys. The authors also noted that the D-dimer levels increased in the setting of diffuse intravascular coagulation associated with shock [28]. Microthrombosis caused by increased active coagulation markers or impaired fibrinolysis is considered to cause acute myocardial damage by affecting the coronary microvascular bed and D-dimer elevation is associated with poor clinical outcomes. In addition, D-dimer levels are considered to be associated with a high risk of thrombosis [28,29]. In this study, we presented that platelet levels decreased and D-dimer and INR levels increased progressively in severe COVID-19 patients.

Critically ill patients with COVID-19 are at risk of developing a hypercoagulable condition with high mortality risk. It is recommended to use prophylactic, intermediate, or therapeutic doses of anticoagulants for thromboprophylaxis in hospitalized COVID-19 patients [30]. The exact cause of bleeding in COVID-19 patients is not clear. Questions arise as to whether the underlying cause of elevated D-dimer levels, thrombotic events, and bleeding is coagulopathy caused by a pathophysiologically different infection or activation of the coagulation system in the setting of severe inflammation. In a recent systematic review and meta-analysis, the incidence of major bleeding was 3.9% (95% CI: 1.2-7.9) [31]. It has been reported that the overall risk of bleeding among hospitalized COVID-19 patients is 4.8%, and this risk increases to 7.6% in critically ill patients [32]. In a recent study, major bleeding was found in 5.7% of hospitalized patients with COVID-19 receiving intermediate or therapeutic-intensity anticoagulant therapy. The use of therapeutic-intensity anticoagulation at admission, critical illness, and high D-dimer or ferritin levels has been associated with an increased risk of major bleeding [33]. In the previous studies, hospitalized patients due to COVID-19 were evaluated in terms of bleeding, and in our study, patients who died from COVID-19 were evaluated. In our study, we determined age, HT, DM, use of ASA or LMWH, hemoglobin, D-dimer, INR, and AKI as independent predictors of major bleeding. Although the study design did not give a clear idea of the effect of bleeding on mortality, the high prevalence of bleeding in patients who died during hospitalization may have indirectly revealed the relationship between worse clinical outcomes and bleeding. Therefore, we consider that the dose of anticoagulant therapy should be adjusted on a patient basis to improve the prognosis of the disease in comorbid patients with high bleeding risk. Moreover, perhaps, a COVID-19-specific scoring system consisting of clinical, laboratory, and imaging parameters could be established to better identify individuals at high risk of bleeding.

Our study had several limitations. First, only patients who died of COVID-19 were included in the study and thus the results of the study only reflect the patients with severe and critical symptoms and may not be generalized to most of the COVID-19 patients who typically have mild or moderate symptoms. Second, most of our patients had a poor general clinical condition and consciousness at the time of admission and thus there may be missing data in their patient histories. Third, since the doses of LMWH for anticoagulation applied to the patients at the beginning and during the follow-up were different, a comparison could not be performed as a prophylactic, intermediate, or full dose. Fourth, since COVID-19 is a multisystemic disease, it was difficult to pinpoint the exact role of bleeding among the definitive causes of death. Autopsy studies, therefore, could fill this gap. Finally, due to the risk of infection, COVID-19 patients were not adequately investigated for intracranial events, pulmonary embolism, and peripheral embolic events with computed tomography and Doppler ultrasonography.

## CONCLUSION

Our results showed that a high proportion of COVID-19 non-survivors suffered from major bleeding complications. Anticoagulation therapy may increase the risk of major and potentially fatal bleeding, as well as be effective in the prevention and treatment of venous thromboembolism in COVID-19 patients. The results obtained in this study suggest that major bleeding is common in patients who die of COVID-19. Early identification of patients with comorbid conditions and at risk of developing major bleeding on anticoagulant therapy may be important to improve outcomes. Large-scale, comprehensive, and prospective studies are thus needed to determine the optimal heparin dose and how long treatment should be continued.
